# Authors' reply to: Pre-ICU length of stay in very old patients: interpreting prognosis within the ICU admission pathway

**DOI:** 10.1016/j.aicoj.2026.100135

**Published:** 2026-08-17

**Authors:** Björn Ahlström, Miklos Lipcsey

**Affiliations:** a Anesthesiology and Intensive Care, Department of Surgical Sciences, Uppsala University, Uppsala, Sweden; b Center for Clinical Research Dalarna, Uppsala University, Sweden; c Anesthesiology, Perioperative and Intensive Care, Falun Hospital, Falun, Sweden; d Anesthesiology, Perioperative and Intensive Care, Uppsala University Hospital, Uppsala, Sweden; e Hedenstierna Laboratory, Department of Surgical Sciences, Uppsala University, Uppsala, Sweden

To the Editor,

We thank Zhou and Hua for a close reading [[1], [2]]. The correspondence questions the admission-route denominators and the restriction to ICU-admitted patients. Both points can be answered with data.

The admission-route denominator. The route percentages in Table 1 of the original article use available-case denominators. Route was recorded for 192 410 of 315 042 patients (61.1 %), so the 98 685 direct-from-emergency-room (ER) admissions are 51.3% of those with a recorded route and 31.3 % of the full cohort. For patients 80 years or older, 44.9% and 29.3 %. The 51 % cited in our Discussion needs the same qualification. Table 1 below presents route composition by pre-ICU length of stay (LOS) and age decile, with full-cohort denominators.

**Table 1:** Admission route by pre-ICU hospital length of stay and age decile, with the full cohort as denominator.

	18−29	30−39	40−49	50−59	60−69	70−79	≥80	Overall
Pre-ICU LOS 0 days, N	23 814	14 759	18 274	24 009	34 557	33 848	25 504	174 765
Admission from								
Emergency room	13 135 (55.2%)	6 881 (46.6%)	9 218 (50.4%)	11 715 (48.8%)	17 105 (49.5%)	16 645 (49.2%)	13 075 (51.3%)	87 774 (50.2%)
Ward and other in-hospital	2 284 (9.6%)	1 946 (13.2%)	2 275 (12.4%)	3 599 (15.0%)	6 483 (18.8%)	7 051 (20.8%)	4 794 (18.8%)	28 432 (16.3%)
Unknown	8 395 (35.3%)	5 932 (40.2%)	6 781 (37.1%)	8 695 (36.2%)	10 969 (31.7%)	10 152 (30.0%)	7 635 (29.9%)	58 559 (33.5%)

Pre-ICU LOS 1 day, N	3 497	3 351	4 662	8 920	17 658	18 645	10 070	66 803
Admission from								
Emergency room	1 449 (41.4%)	894 (26.7%)	1 118 (24.0%)	1 458 (16.3%)	1 915 (10.8%)	1 814 (9.7%)	1 456 (14.5%)	10 104 (15.1%)
Ward and other in-hospital	1 260 (36.0%)	1 456 (43.4%)	1 635 (35.1%)	2 891 (32.4%)	6 428 (36.4%)	7 062 (37.9%)	4 483 (44.5%)	25 215 (37.7%)
Unknown	788 (22.5%)	1 001 (29.9%)	1 909 (40.9%)	4 571 (51.2%)	9 315 (52.8%)	9 769 (52.4%)	4 131 (41.0%)	31 484 (47.1%)

Pre-ICU LOS 2–3 days, N	1 139	1 303	1 767	3 137	6 659	8 007	6 127	28 139
Admission from								
Emergency room	36 (3.2%)	22 (1.7%)	20 (1.1%)	61 (1.9%)	120 (1.8%)	110 (1.4%)	102 (1.7%)	471 (1.7%)
Ward and other in-hospital	789 (69.3%)	913 (70.1%)	1 067 (60.4%)	1 855 (59.1%)	4 148 (62.3%)	5 155 (64.4%)	4 032 (65.8%)	17 959 (63.8%)
Unknown	314 (27.6%)	368 (28.2%)	680 (38.5%)	1 221 (38.9%)	2 391 (35.9%)	2 742 (34.2%)	1 993 (32.5%)	9 709 (34.5%)

Pre-ICU LOS 4 days or more, N	1 282	1 345	2 404	5 445	12 001	14 450	8 408	45 335
Admission from								
Emergency room	25 (2.0%)	21 (1.6%)	28 (1.2%)	42 (0.8%)	80 (0.7%)	78 (0.5%)	62 (0.7%)	336 (0.7%)
Ward and other in-hospital	784 (61.2%)	756 (56.2%)	1 193 (49.6%)	2 315 (42.5%)	5 412 (45.1%)	6 931 (48.0%)	4 728 (56.2%)	22 119 (48.8%)
Unknown	473 (36.9%)	568 (42.2%)	1 183 (49.2%)	3 088 (56.7%)	6 509 (54.2%)	7 441 (51.5%)	3 618 (43.0%)	22 880 (50.5%)

Values are n (% of the age by length of stay cell). Ward and other in-hospital comprises ward and other locations (57 979 in the full cohort), operating theatre (24 259), other ICU (6 317), intermediate care unit (3 702) and recovery room (1 468). Unknown denotes patients for whom the SAPS3 Box I record, and therefore admission route, was not reported.

Curves excluding direct-from-ER admissions. Route is a SAPS3 item, and missing route is a strict subset of missing SAPS3. Missingness fell from nearly 100% before 2008 to 10–12% from 2012, and ranged from 26.4 % in district to 54.7 % in university hospitals. This follows registry uptake and reporting practice, not patient characteristics. Such curves require a recorded route, which removes the first three calendar years and reweights the cohort toward later years and reporting units. Neither that restriction nor imputation is defensible here.

The composition of day 0. Day 0 is not a single clinical starting point. Thirty-day mortality at day 0 was almost identical across routes: in patients 80 years or older, 35.8% the ER, 34.8% for ward and other in-hospital locations, and 34.5% where route was unrecorded. Within ER admissions, mortality changed little from day 0 to day 1, 35.8% to 33.7%. Route does not appear to generate the early non-monotonicity, although day 0 includes 4 324 patients (2.5%) with no qualifying National Patient Registry episode, assigned zero days on the assumption that this registry is complete.

Surgical status does separate the strata. Among patients with a recorded route, medical admissions were 89.5% of day 0 but 63.7% of day 1, while elective surgery rose from 3.1% to 16.4%. Crude mortality from day 0 to day 1 was flat in medical admissions in every decile, 35.8% to 36.2% in the oldest, and fell in surgical ones, 38.8 % to 32.8 % for acute surgery. The most urgent acute surgical patients reach the ICU on the day of admission, and days 1–3 hold low-risk elective preoperative admissions. The physiological mechanism proposed in our Discussion is not excluded, since ER medical admissions fell from 35.7 % to 33.6 % in the oldest decile, but case mix dominates. These unadjusted proportions, qualify rather than replace the model estimates and surgical status was not in our *a priori* adjustment set, a limitation of the early curve.

Cohort entry. We agree. Our estimates describe ICU-admitted patients, and evaluating pre-ICU LOS as an input to admission decisions would require a population defined at referral or assessment.

Sincerely,

Björn Ahlström and Miklos Lipcsey

## Authors' contributions

BA and ML conceptualized the reply, BA analyzed the data, BA wrote a first draft of the manuscript, and all authors revised the text and approved the final draft.

## Consent for publication

Not applicable.

## Ethics approval and consent to participate

The study was approved by the Regional Ethics Committee of Uppsala (DNR 2016/421) and consent was waived.

## Declaration of Generative AI and AI-assisted technologies in the writing process

During the preparation of this work, the authors used Claude AI in order to check and debug R code and for language editing of the manuscript. After using this tool, the authors reviewed and edited the content as needed and take full responsibility for the content of the published article.

## Funding

The Akademiska University Hospital and the healthcare Region Dalarna funded this research. The funding sources had no role in the design and conduct of the study; the collection, management, analysis, or interpretation of the data; the preparation, review, or approval of the manuscript; and the decision to submit the manuscript for publication.

## Availability of data and material

The data that support the findings of this study are available from the registry holders (the Swedish Intensive Care Registry and the Swedish Board of Health and Welfare), but restrictions apply to the availability of these data, which were used under license for the current study, and so are not publicly available. Data are, however, available from the authors upon reasonable request and with permission of the Swedish Ethical Review Authority and Uppsala University, under the limitations of the European General Data Protection Act.

## Declaration of competing interest

The authors declare that they have no competing interests
